# The relationship between cumulative dose of immunosuppressive agents and COVID‐19‐associated mucormycosis: A multicenter cross‐sectional study

**DOI:** 10.1002/hsr2.950

**Published:** 2022-11-22

**Authors:** Mohsen Rastkar, SeyedAhmad SeyedAlinaghi, Behzad Asanjarani, Goli Siri, Hamed Abdollahi, Ladan Ghadami, Mehrdad Hasibi, Rozita Khodashahi, AmirBehzad Bagheri, Ali Asadollahi‐Amin

**Affiliations:** ^1^ Students’ Scientific Research Center, Tehran University of Medical Sciences Tehran Iran; ^2^ Iranian Research Center for HIV/AIDS, Iranian Institute for Reduction of High‐Risk Behaviors Tehran University of Medical Sciences Tehran Iran; ^3^ Department of Internal Medicine, Amir Alam Hospital Tehran University of Medical Sciences Tehran Iran; ^4^ Department of Anesthesia and Critical Care, Amir Alam Hospital Complexes Tehran University of Medical Sciences Tehran Iran; ^5^ Department of Health Care Management, Amir Alam Hospital Complexes Tehran University of Medical Sciences Tehran Iran; ^6^ Clinical Research Development Unit, Imam Reza Hospital, Faculty of Medicine Mashhad University of Medical Sciences Mashhad Iran; ^7^ Student Research Committee, Faculty of Medicine Mashhad University of Medical Sciences Mashhad Iran; ^8^ Division of Vascular Surgery and Endovascular Therapy, Michael E. DeBakey Department of Surgery Interdisciplinary Consortium on Advanced Motion Performance, Baylor College of Medicine Houston Texas USA

**Keywords:** corticosteroids, COVID‐19, immunosuppressive, mucormycosis, outcome, tocilizumab

## Abstract

**Background and Aims:**

Immunosuppressive therapy has a key role in developing coronavirus disease‐2019 (COVID‐19)‐associated mucormycosis. In this study, we investigated the effect of the type and cumulative dose of immunosuppressive agents on COVID‐19‐associated mucormycosis.

**Methods:**

We designed a descriptive cross‐sectional study involving three COVID‐19 hospitals in Iran. Clinical and demographic data were gathered from the medical records and checked by two independent researchers to minimize errors in data collection.

**Results:**

Seventy‐three patients were included in the study. The mean age of cases was 57.41 (SD = 12.64) and 43.8% were female. Among patients, 20.5% were admitted to the intensive care unit (ICU) during COVID‐19. Furthermore, 17 patients (23.29%) had a history of diabetes mellitus. Sixty‐nine patients (94.52%) had a history of receiving corticosteroids (dexamethasone) during treatment of COVID‐19, and of those, five patients (6.85%) received Tocilizumab beside. The mean cumulative dose of corticosteroids prescribed was 185.22 mg (SD = 114.738). The average cumulative dosage of tocilizumab was 720 mg (SD = 178.89). All of the included patients received amphotericin B for mucormycosis treatment, and 42 survived (57.53%). Also, there was a significant relationship between hospitalization in ICU for COVID‐19 and the mucormycosis outcome (*p* = 0.007). However, there weren't any significant associations between cumulative doses of immunosuppressive drugs and mucormycosis outcome (*p* = 0.52).

**Conclusion:**

The prevalence of COVID‐19‐associated mucormycosis is increasing and should be considered in the treatment protocols of COVID‐19. Controlling risk factors such as diabetes, malignancy and the administration of immunosuppressive agents based on recommended dosage in validated guidelines are ways to prevent mucormycosis.

## 1 BACKGROUND

Coronavirus disease‐2019 (COVID‐19) is an acute respiratory disease caused by the severe acute respiratory syndrome coronavirus 2 (SARS‐CoV‐2), which has been responsible for a global pandemic since 2020. Patients with COVID‐19 present with a variety of symptoms, the most prevalent of which are fever, cough, fatigue, and loss of smell and taste.^1^ Although the majority of COVID‐19 cases experience mild illness, patients with risk factors such as older age, cardiovascular disease, chronic respiratory disease, and diabetes are prone to severe disease that requires advanced therapeutic interventions.^2^ Additionally, several secondary infections, including bacterial and fungal infections, have been observed in COVID‐19 patients, which exacerbated the complications of the disease. Of those, mucormycosis has raised concerns due to its high incidence and poor outcome.^3^

Mucormycosis is an acute but rare fungal opportunistic disease caused by the Mucormycetes group, including *Mucor*, *Rhizopus*, *Rhizomucor*, *Apophysomyces*, and *Abdidia*.^4^ It mostly involves patients with underlying conditions such as uncontrolled diabetes, immunodeficiency, and organ transplantation.^5^ Mucormycosis is associated with invasive tissue necrosis and infarction and is more common in the rhino‐orbital‐cerebral type.^6^ Due to the high rate of disease progression and its high mortality, examining the risk factors affecting the incidence and control of mucormycosis owns of great importance.

Immunosuppressive therapy is one of the risk factors that could be crucial in developing COVID‐19‐associated mucormycosis. In the procedure of COVID‐19 treatment, a variety of immunosuppressive medications are used in varying doses to reduce the symptoms of the host immune response and cytokine storm.^7^ This includes a number of medications such as corticosteroids, baricitinib, and tocilizumab (Actemra). Thus, by examining the impact of different medications and doses used to suppress the immune system on the risk of developing mucormycosis, immunosuppressive medications in COVID‐19 patients might be prescribed with greater prudence. Therefore, in this study, we investigated the effect of the type and cumulative dose of immunosuppressive agents on COVID‐19‐associated mucormycosis.

## 2 METHODS

### 2.1 Study design

We performed a descriptive cross‐sectional study involving three COVID‐19 hospitals in Iran, including two hospitals in Tehran (Amir Alam hospital, Imam Khomeini Hospital complex) and one hospital in Mashhad (Imam Reza hospital). We collected data on all confirmed cases of mucormycosis from March 2021 until December 2021. This study followed the Declaration of Helsinki and was approved by the ethics committee of Tehran University of medical sciences (IR.TUMS.IKHC.REC.1400.491).

### 2.2 Study subjects and definitions

Patients were included in the study based on the following criteria: (1) Positive diagnosis of COVID‐19 based on computed tomography (CT), real‐time reverse transcriptase‐polymerase chain reaction (RT‐PCR), or rapid antigen test. (2) Positive diagnosis of mucormycosis based on histopathological examination. (3) The interval between the diagnosis of COVID‐19 and mucormycosis was equal or less than 3 months.

Histopathological criteria for diagnosis of mucormycosis were infiltration of fungal hyphae and neutrophils in tissue, hemorrhage, thrombosis, and tissue infarction. The criteria of intensive care unit (ICU) admission for COVID‐19 were defined based on The National Institutes of Health (NIH) guidelines for COVID‐19.^8^

Clinical and demographic data were gathered from the medical records and checked by two independent researchers to minimize errors in data collection. Patients were informed that their demographic and clinical information would be used in the study, and written informed consent was obtained. The cumulative dose of immunosuppressive therapy was defined as the total dose of any immunosuppressive drug received for the treatment of COVID‐19.

### 2.3 Statistical methods

Data were gathered into an Excel spreadsheet and then transferred to SPSS Statistics 26.0 (IBM, Inc). Descriptive analysis was performed, including frequencies, mean, and standard deviation (SD). Also, bivariate analysis was conducted with the Chi‐square test and independent‐sample *T*‐test.

## 3 RESULTS

Initially, 111 patients with COVID‐19‐associated mucormycosis were included in the study; however, owing to the unavailability of data on the course of COVID‐19; finally, 73 patients were included in the study. The mean age of cases was 57.41 ± 12.64% and 43.8% were female. Among patients included in the study, 20.5% were admitted to the intensive care unit (ICU) during COVID‐19. Furthermore, 17 patients had a history of diabetes mellitus. Sixty‐nine patients had a history of receiving corticosteroids (dexamethasone) during treatment of COVID‐19, and of those, five patients received Tocilizumab beside. The mean cumulative dose of corticosteroids prescribed was 185.22 mg ± 114.738. The average cumulative dosage of Tocilizumab was 720 mg ± 178.89. All of the included patients received amphotericin B for mucormycosis treatment, and 42 survived. Table 1 shows the summary of the characteristics of patients. In addition, the demographic and clinical characteristics of patients based on mucormycosis outcome were compared in Table 2, which indicates a significant relationship between hospitalization in ICU for COVID‐19 and the mucormycosis outcome (*p* = 0.007). However, there weren't any significant differences in cumulative doses between two groups of survivors and non‐survivors of mucormycosis (Table 3).

**Table 1:** Demographic and clinical characteristics of patients

Demographics	Gender, female		32 (43.8%)
	Age (mean (SD))		57.41 (12.64)
Risk factors	Diabetes		17 (23.3%)
	Immunosuppressive therapy	Corticosteroid	69 (94.5%)
		Tocilizumab	5 (6.8%)
	Malignancy		1 (1.37%)
Cumulative dose of immunosuppressive therapy	Corticosteroid (mean (SD))		185.22 (114.74)
	Tocilizumab (mean (SD))		720 (178.89)
COVID‐19 severity	ICU		15 (20.5%)
Antifungal therapy	Amphotericin B		73 (100%)
Outcome	Died		31 (42.5%)
	Survived		42 (57.5%)

Abbreviations: COVID‐19, coronavirus disease‐2019; ICU, intensive care unit; SD, standard deviation.

**Table 2:** Comparison of characteristics of patients based on mucormycosis outcome

	Alive	Died	*p*‐value
Age > 60	18 (42.86%)	16 (51.61%)	0.46
Gender(female)	19 (45.24%)	13 (41.94%)	0.78
diabetes	12 (28.57%)	5 (16.13%)	0.21
corticosteroid	39 (92.86%)	30 (96.77%)	0.47
ICU	4 (9.52%)	11 (35.48%)	0.007
Tocilizumab	3 (7.14%)	2 (6.45%)	0.91

Abbreviations: ICU, intensive care unit; SD, standard deviation.

**Table 3:** Mean difference of immunosuppressant cumulative dose between survived and nonsurvived patients

	Alive Mean (SD)	Died Mean (SD)	*df*	*t*	*p*‐value
Corticosteroid	177.33 (110.37)	195.47 (121.31)	67	0.648	0.52
Tocilizumab	666.67 (230.94)	800 (0)	3	0.775	0.50

## 4 DISCUSSION

As of the time of writing this article, COVID‐19 has infected more than 500 million people and caused the death of about 6 million patients.^9^ Among hospitalized COVID‐19 patients, mucormycosis, as a secondary infection, has had a prevalence of seven cases per 1000 patients which is 50 times higher than its prevalence among diabetic patients (0.14 cases per 1000).^10^ The mortality rate of mucormycosis is about 46%, and a delay in diagnosis only for a week will increase the risk of death from 35% to 66%, which can be prevented by timely diagnosis.^11^, ^12^

In our study, 42.5% of patients with mucormycosis lost their lives, demonstrating a high risk of death in COVID‐19‐associated mucormycosis. In addition, 18 individuals had a history of underlying conditions such as diabetes mellitus and malignancy. As a result, managing underlying diseases could be an essential factor in reducing the risk of mucormycosis.

Moreover, 69 patients had a history of receiving immunosuppressive drugs, which shows a high risk of administrating these medications for secondary infections in the treatment of COVID‐19. The average cumulative dose of corticosteroids prescribed for patients in the COVID‐19 period was 185.22 mg. The type of corticosteroid received in all patients was dexamethasone. The NIH guideline for COVID‐19 treatment recommends prescribing 6 mg of dexamethasone daily intravenously for 10 days or until the end of the hospital course, whichever occurs first.^8^ Therefore, it seems most patients have received high doses of corticosteroids in this study, which may have contributed to the increased risk of developing mucormycosis. Another drug prescribed for immunosuppressive therapy was Tocilizumab, which was used in only five patients. Tocilizumab is a recombinant monoclonal antibody; that acts against interleukin‐6 receptors as a pro‐inflammatory cytokine.^13^ Based on the NIH guideline, Tocilizumab should be used in a single dose of 8 mg/kg up to a maximum dosage of 800 mg.^8^ Thus, Tocilizumab prescription for the patients included in the study was in accordance with the NIH guideline.

Another important point is the type of drug used to suppress the immune system. In our study, all people who received immunosuppressive therapy took dexamethasone as a corticosteroid, which could be due to its lower cost, availability, and better efficacy.^14^ As the drug shortage, only five people received tocilizumab along with dexamethasone. Due to the limited use of non‐corticosteroid immunosuppressive medications, no precise conclusions could be drawn about the relationship between drug type and mucormycosis. In 2020, Patel et al.^15^ In a retrospective study examined 287 patients with mucormycosis, 187 of whom were previously infected with COVID‐19. Among COVID‐19 patients, 146 patients used dexamethasone, and five cases used Tocilizumab for treatment.^15^ Also, in a systematic review of case reports and case series of 101 patients with mucormycosis, 76.3% of patients have used corticosteroids and 4.1% have used both Tocilizumab and corticosteroids for the treatment of COVID‐19.^16^ In a cross‐sectional study, Dubey et al. examined the risk factors for mucormycosis in 55 patients with COVID‐19. The study showed corticosteroids were used for treatment in 60% of patients.^17^ Therefore, similar to our study, in most COVID‐19 patients, corticosteroids have been used for immunosuppressive therapy, and different types of drugs cannot be compared accurately.

In COVID‐19, there is a significant reduction in the number of clusters of differentiation 4 (CD4) and CD8 cells.^18^ Therefore, this immunosuppression could increase the risk of secondary infections. Previous studies have reported that a significant result of hyperglycemia is increased expression of glucose‐regulated protein (GRP78) expression. This protein is located in the endoplasmic reticulum of mammalian cells and could act as a receptor for Rhizopus spore coat protein homologs (CotH) protein kinase. Thus, it facilitates the attachment of the fungus particle to endothelial cells.^19^ The key point of this finding is that the expression of GRP78 in COVID‐19 patients has increased compared to controls, which may indicate that the SARS‐CoV‐2 virus, regardless of the immunosuppressive therapy, can be a risk factor for developing mucormycosis.^20^

Another finding of our study was the association between the severity of COVID‐19 disease and the outcome of mucormycosis. Due to the limited available information, the severity of COVID‐19 was considered based on hospitalization in the ICU, which shows an increased likelihood of severe mucormycosis and death outcomes. However, this finding may indicate that people admitted to the ICU for COVID‐19 have certain risk factors that may affect the outcome of mucormycosis, which might be due to higher doses of immunosuppression, although it was not evaluated in this study.

The incompleteness of data in medical records and lack of microbiological diagnosis of mucormycosis were our main limitations. Furthermore, the limited use of non‐corticosteroid immunosuppressive medications: Tocilizumab, baricitinib, tofacitinib, and sarilumab made it difficult to draw accurate conclusions about the effect of the type of drug on the risk of mucormycosis.

## 5 CONCLUSION

the prevalence of COVID‐19‐associated mucormycosis is increasing and should be considered in the treatment protocols for COVID‐19. Controlling risk factors such as diabetes, malignancy, organ transplant, and the administration of immunosuppressive agents based on recommended dosage in validated guidelines are ways to prevent mucormycosis. However, the risk factors for mucormycosis in COVID‐19 patients should be further investigated in future studies to decrease the risk of developing mucormycosis among COVID‐19 patients.

## AUTHOR CONTRIBUTIONS

**Mohsen Rastkar**: Validation; writing – original draft; writing – review & editing. **SeyedAhmad SeyedAlinaghi**: Methodology; software; supervision. **Behzad Asanjarani**: Data curation. **Goli Siri**: Data curation; validation. **Hamed Abdollahi**: Data curation. **Ladan Ghadami**: Formal analysis. **Mehrdad Hasibi**: Investigation; visualization. **Rozita Khodashahi**: Conceptualization; investigation; visualization. **AmirBehzad Bagheri**: Conceptualization; writing – original draft. **Ali Asadollahi‐Amin**: Supervision; writing – original draft; writing – review & editing.

## CONFLICTS OF INTEREST

The authors declare no conflicts of interest.

## CONSENT FOR PUBLICATION

All authors have read and approved the final version of the manuscript corresponding author had full access to all of the data in this study and takes complete responsibility for the integrity of the data and the accuracy of the data analysis. This article has not been published and is not under consideration for publication elsewhere.

## ETHICS STATEMENT

We also declare that the study was performed according to international, national, and institutional rules considering animal experiments, clinical studies, and biodiversity rights.

## TRANSPARENCY STATEMENT

The lead author Ali Asadollahi‐Amin affirms that this manuscript is an honest, accurate, and transparent account of the study being reported; that no important aspects of the study have been omitted; and that any discrepancies from the study as planned (and, if relevant, registered) have been explained.

## Data Availability

All information provided in this article can be obtained from the author on request.
